# Patient-Related Determinants of Glycaemic Control in People with Type 2 Diabetes in the Gulf Cooperation Council Countries: A Systematic Review

**DOI:** 10.1155/2018/9389265

**Published:** 2018-02-25

**Authors:** Mohammed J. Alramadan, Afsana Afroz, Sultana Monira Hussain, Mohammed Ali Batais, Turky H. Almigbal, Hassan Ahmad Al-Humrani, Ahmed Albaloshi, Lorena Romero, Dianna J. Magliano, Baki Billah

**Affiliations:** ^1^Department of Epidemiology and Preventive Medicine, Monash University, Melbourne, VIC, Australia; ^2^College of Medicine, King Saud University, Riyadh, Saudi Arabia; ^3^Diabetes Centre, Directorate of Health Affair, Hofuf, Saudi Arabia; ^4^Diabetes Centre, Directorate of Health Affair, Jeddah, Saudi Arabia; ^5^The Ian Potter Library, The Alfred, Melbourne, VIC, Australia; ^6^Baker Heart and Diabetes Institute, Melbourne, VIC, Australia

## Abstract

The aim of this systematic review is to assess patient-related factors affecting glycaemic control among people with type 2 diabetes in the Arabian Gulf Council countries. MEDLINE, Embase, PsycINFO, CINAHL, and Cochrane CENTRAL databases were searched from their date of inception to May 2016. Two researchers independently identified eligible studies and assessed the risk of bias. A total of 13 studies met the inclusion criteria. One study was population based, six recruited participants from multiple centres, and the remaining were single centred. The majority of the studies were of low to moderate quality. Factors associated with poor glycaemic control include longer duration of diabetes, low level of education, poor compliance to diet and medication, poor attitude towards the disease, poor self-management behaviour, anxiety, depression, renal impairment, hypertension, and dyslipidaemia. Healthcare providers should be aware of these factors and provide appropriate education and care especially for those who have poor glycaemic control. Innovative educational programs should be implemented in the healthcare systems to improve patient compliance and practices. A variation in the results of the included studies was observed, and some potentially important risk factors such as dietary habits, physical activity, family support, and cognitive function were not adequately addressed. Further research is needed in this area.

## 1. Introduction

Diabetes mellitus is one of the major public health issues of the 21st century [1–3]. Globally, 8.8% (415 million) of adults suffered from diabetes in 2015, and it is estimated that 652 million people (10.4%) will have diabetes by 2040 [2]. The World Health Organization (WHO) reported that high blood glucose level due to diabetes is the third highest risk factor for premature mortality after high blood pressure and tobacco use [4]. Diabetes is attributed to 14.5% of all-cause mortality among adults, and half of these deaths occur in adults under the age of 60 years [2]. Nonetheless, diabetic complications are a major cause of disability and reduced quality of life. The estimated total global health expenditure due to diabetes is $673 billion in 2015, and it will reach $802 billion in 2030 [2].

A recent International Diabetes Federation (IDF) report suggests that the Middle East and North Africa regions, which include the Gulf Cooperation Council (GCC) countries (Bahrain, Kuwait, Oman, Qatar, Saudi Arabia, and the United Arab Emirates (UAE)), have the highest prevalence of diabetes (10.7%) in the world next to North America and the Caribbean region (11.5%) [2]. An estimated 35.4 million adults in the Middle East and North Africa regions had diabetes in 2015, of whom over 40.6% were undiagnosed [2]. The number of people with diabetes in this region is anticipated to reach 72.1 million by 2040 [2]. The GCC countries share boundaries and similar climates, cultures, lifestyles, and economic development. These countries are among those with the highest prevalence of diabetes in the Middle East and North Africa regions and globally, with the prevalence of the disease ranging from 14.8% (Oman) to 20% (Saudi Arabia, Kuwait, and Qatar) [2].

Diabetes management involves strictly maintaining a person's blood glucose level close to the normal range. There is a strong relationship between an elevated blood glucose level and the risk of complications and mortality in people with diabetes [5, 6]. Poor glycaemic control is defined as a glycated haemoglobin (HbA1c) equal to or above 7% or a fasting plasma sugar (FPS) above 7.2 mmol/l for adults who are not pregnant [7]. Poor glycaemic control (HbA1c > 7%) among people with type 2 diabetes mellitus (T2DM) in the GCC countries is common, ranging between 59% and 70.7% in Saudi Arabia [8, 9], 68% and 69% in the UAE [10, 11], 86.5% and 88.8% in Bahrain [12, 13], and 54% and 65% in Oman [14, 15], while a study reported that 55% of people with T2DM in Kuwait have HbA1c > 9% [16].

A number of studies have assessed factors associated with poor glycaemic control among people with T2DM in GCC countries sporadically. A systematic approach is needed to summarise their findings in order to identify gaps in the literature and provide guidelines for future research. Thus, the aim of this systematic review is to assess patient-related factors that affect glycaemic control among people with type 2 diabetes in the GCC countries.

## 2. Methods

### 2.1. Literature Search Strategy

A senior librarian (LR), with input from the research team, developed and implemented a comprehensive search using Embase, CINAHL, Cochrane Library, MEDLINE, and PsycINFO from the date of their inception to the 31 of May 2016. The search covered three concepts: T2DM, glycaemic control, and the Arabian Gulf Council countries. For T2DM, the following terms were used in the search combined by “OR”: “diabetes mellitus, type 2” (subject headings [SH]), Hyperglycaemic (SH), “adult-onset diabet^∗^,” “ketosis-resistant diabet^∗^,” “maturity-onset diabet^∗^,” “non-insulin-dependent diabet^∗^,” “noninsulin-dependent diabet^∗^,” “slow-onset diabet^∗^,” “type 2 diabet^∗^,” “type ii diabet^∗^,” “insulin resistance,” “insulin resistant,” and “T2D.” For glycaemic control, the following terms were used in the search combined by “OR”: “glucose,” “blood sugar,” “glyco^∗^,” “glyca^∗^,” “glyce^∗^,” “hb a1^∗^,” “hba1^∗^,” “haemoglobinA1^∗^,” “hemoglobinA1^∗^,” “haemoglobin A1^∗^,” and “hemoglobin A1^∗^.” For searching the Arabian Gulf countries, the following terms were used combined by “OR”: “Saudi^∗^,” “Kuwait^∗^,” “Bahrain^∗^,” “Qatar^∗^,” “Arab^∗^,” “Emirat^∗^,” “Oman^∗^,” “Middle East^∗^,” “Gulf cooperation,” and “gulf co-operation.” The final search was then conducted after having combined the three concepts using “AND.” The search was not limited by language. Relevant articles were also sought by searching the reference lists of articles retrieved for full-text review.

### 2.2. Selection of Studies

Two reviewers (MJA and AA) independently screened the retrieved articles by title, then by abstract, and finally by reviewing the full text of eligible articles. Any disagreement between the two reviewers was resolved by discussion with a third reviewer (BB).

### 2.3. Studies Inclusion and Selection Criteria

All observational studies that examined the effect of patient-related factors affecting glycaemic control among adults with T2DM living in the GCC countries were considered for inclusion. Because the majority of people in the GCC countries have T2DM, studies that assessed the level of control among people with diabetes in general (types 1 and 2) were included. Studies were excluded if they did not include adults with T2DM, if the sample was recruited from outside of the GCC countries, and/or if the study did not examine the association between patient-related factors and glycaemic control. Studies that focused only on type 1 diabetes, children with T2DM, gestational diabetes, or adults with impaired glucose tolerance or impaired fasting glucose were excluded. Studies investigating the effectiveness of antidiabetic medications only were also excluded. In addition, articles were excluded if they were reviews or conference presentations.

### 2.4. Data Extraction

Two reviewers (MJA and AA) independently extracted the data using a data extraction form. The extracted data include the first author, the year of publication, the name of the country, the sampled population, the number of participants, participants' gender, participants' age, the method of assessing glycaemic control, the data collection tool, the risk factors investigated, and the factors found to be associated with glycaemic control.

### 2.5. Assessment of Quality

Two reviewers (MJA and AA) independently assessed the risk of bias of the included studies using the National Heart Lung and Blood Institute (NHLBI) quality assessment tool for observational studies (Tables 1 and 2) [17]. The tool assesses the internal validity and risk of bias using 14 criteria for cohort and cross-sectional studies and 12 criteria for case-control studies. Each criterion was rated as “yes,” “no,” “cannot determine,” “not applicable,” or “not reported.” An overall judgment of the quality of the study was then rated as high (low risk of bias), fair (moderate risk of bias), or low (high risk of bias). Any disagreement between the two reviewers regarding the assessment of risk of bias was settled by discussion.

## 3. Results

### 3.1. Search Results

**Figure 1:**
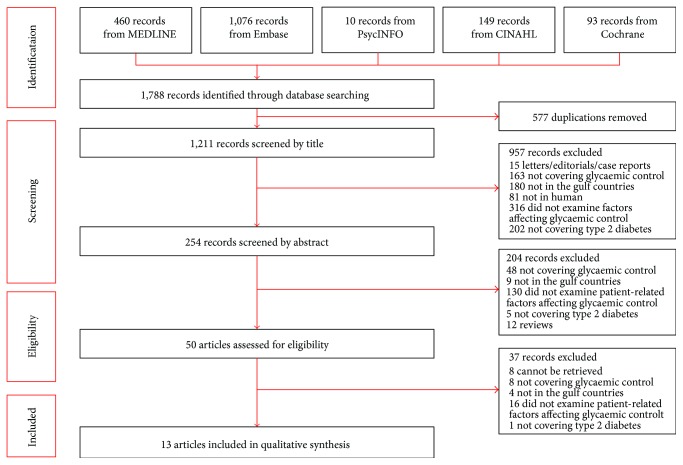
Flow chart of the systematic literature search.

Figure 1 shows a flow chart of the search results and the number of studies included in this review. A total of 1788 articles were retrieved from the five databases (MEDLINE = 460, Embase = 1076, PsycINFO = 10, CINAHL = 149, and Cochrane = 93). After the removal of duplicates, 1211 articles remained. After having screened article titles and abstracts, 1161 articles were removed as these were letters, editorials, case reports, review articles, animal/cell studies, not from the GCC countries, did not include adult participants with T2DM, did not cover glycaemic control, or did not assess factors affecting glycaemic control. Thus, 50 full-text articles were screened for further eligibility. Of these 50 articles, 37 articles were excluded. Eight articles did not evaluate glycaemic control, four studies were not conducted in the GCC countries, one study did not include people with T2DM, and 16 did not evaluate patient-related factors affecting glycaemic control. In addition, eight articles were conference papers and were therefore excluded. A total of 13 studies met the inclusion criteria of this review.

### 3.2. Characteristics of Included Studies

The characteristics of the included studies are described in Table 3. Five studies were from Saudi Arabia [8, 18–21], four from Oman [14, 15, 22, 23], three from the UAE [10, 11, 24], and one from Bahrain [25]. Only one study was population based [20], while three studies recruited participants from multiple primary healthcare centres regulated by one hospital (including the participants of this hospital) [10, 11, 24] and three studies recruited participants from a number of primary healthcare centres [22, 23, 25]. Of the remaining six studies, four recruited participants from a single hospital [8, 15, 18, 19], and two recruited participants from a single primary healthcare centre [14, 21]. All studies were cross-sectional [8, 10, 11, 14, 15, 19–25] except for one which was case-control in design [18]. Three studies extracted data from medical records regarding sociodemographic factors, medical history (duration of diabetes, comorbidities, complications, and modality of treatment), and biochemical data related to glycaemic control [14, 21, 22]. In nine studies, data on sociodemographic and medical history were collected using an interviewer-administered questionnaire [8, 10, 11, 15, 18–20, 24, 25], while one study used self-administered questionnaire [23]. In these 10 studies, data regarding glycaemic control were collected by laboratory investigation. Five studies recruited participants with type 1 and 2 diabetes [10, 18–20, 24], while the eight other studies included participants with T2DM only [11, 14, 15, 21–23, 25]. The number of participants per study ranged from 103 to 1266, with a median of 300 participants. Most of the studies included both males and females [8, 10, 11, 14, 15, 18, 20–25], while one study included only female participants [19]. Mean age of participants was reported in nine studies that ranged between 42.6 ± 9.1 and 57.3 ± 14.4 years [8, 10, 11, 14, 19–22, 25]. Two studies reported age as categories: in one study, the age categories ranged from 20 years to +59 years [23], and in the other study, the age categories ranged from 30 years to +60 years [15]. Two other studies did not report the age of the participants [18, 24]. Glycaemic control was assessed using haemoglobin A1c (HbA1c) in 10 studies [8, 10, 11, 14, 15, 18, 19, 22, 23, 25], while two studies used fasting blood glucose (FBG) [21, 24] and one used random blood glucose (RBG) [20].

### 3.3. Quality Assessment (Risk of Bias)

Of the 13 studies included, seven (53.8%) were of low quality, four (50.8%) were of moderate quality, and two (15.4%) were of high quality (Table 4). Seven studies (53.8%) recruited a small sample size with no justification or calculation of power [8, 14, 18, 19, 21, 23, 25]. The sampling was not random in 10 studies (76.9%) [8, 14, 15, 18, 19, 21–25]. The majority of the studies (69.2%) did not adjust for possible confounding factors of the association between glycaemic control and the assessed risk factors [8, 11, 14, 15, 18, 19, 23–25]. In addition, the results were not generalisable in six studies (46.1%) because they were single centred [8, 14, 15, 18, 19, 21].

### 3.4. Key Findings

#### 3.4.1. Nonmodifiable Factors

The results of the 13 studies included in this review are detailed in Table 5. D'Souza et al. found that among elder participants (50–59 years and ≥60 years), larger proportions had poor control compared to younger participants (*p* value <0.001) [15], while Al-Kaabi et al. reported that age was negatively associated with HbA1c level (adjusted beta coefficient −0.023, *p* value 0.047) [10]. In the study by Al-Lawati et al., it was found that, compared to those aged 20–39 years, the adjusted odds ratio (OR) of good glycaemic control was 1.7-fold (*p* value: 0.01) and 2.5-fold (*p* value: 0.001) higher for those aged 40–59 years and ≥60 years, respectively [22]. On the other hand, the remaining seven studies did not find any association [8, 11, 14, 18, 20, 21, 24].

The association between gender and glycaemic control was evaluated in 10 studies. Of these, one study reported that female gender was associated with poor glycaemic control compared to male gender (adjusted OR: 2.84, *p* value <0.05) [21]. In contrast, Al-Lawati et al. showed that female gender was associated with good glycaemic control (adjusted OR: 1.5, *p* value: 0.001) [22]. No association was found between gender and glycaemic control in the other eight studies [8, 10, 11, 14, 15, 18, 20, 24].

Three out of eight studies that assessed the association between the duration of diabetes and glycaemic control have found an association. Binhemd et al. have reported a positive correlation between the duration and HbA1c level (*p* < 0.001) [19]. In the study by Al-Lawati et al., the adjusted OR of good glycaemic control for people with a duration of diabetes ≥5 years was 0.8 (*p* value: 0.041) compared to those with a duration of <5 years [22]. D'Souza et al. reported that the people with poor control were 49.1%, 52.8%, and 70.5% for diabetes duration groups 0–9 years, 10–19 years, and ≥20 years, respectively (*p* value <0.001) [15]. In the other five studies, no association was found [11, 14, 18, 19, 21].

Two studies assessed the effect of family history of diabetes on glycaemic control. In one study, no significant association was found [21]. Meanwhile, in the other study, a family history of diabetes was found to be associated with lower risk of poor glycaemic control (OR: 0.39, *p* value: 0.001) [24]. This study also assessed the effect of ethnicity on glycaemic control, but no association was found.

#### 3.4.2. Modifiable Factors

A number of modifiable factors were found to be associated with glycaemic control. Binhemd et al. reported a negative correlation between HbA1c and patient compliance to management as well as the attitude towards the disease (*p* value < 0.001 and 0.01, resp.) [19]. Al-Hayek et al. showed that compared to participants with HbA1c < 7%, those with HbA1c ≥ 7% had lower mean score of adherence to medication (5.4 ± 1.2 compared to 7.4 ± 1.4, *p* value < 0.001), higher mean score of anxiety (10.3 ± 1.7 compared to 7.9 ± 1.3, *p* value < 0.001), and higher mean score of depression (9.8 ± 1.3 compared to 6.9 ± 0.9, *p* value < 0.001) [8]. Malik et al. found that individuals who were followed up in primary healthcare centres were more likely to have poor glycaemic control (OR: 2.4, *p* value: 0.001) compared to those who were followed up in hospital [24]. Al-Kaabi et al. reported that the number of carbonated drinks (sugar sweetened and sugar free) consumed was positively associated with HbA1c level (adjusted beta coefficient: 0.20, *p* value: 0.029) [10]. Shamsi et al. showed that the mean HbA1c level increases progressively from 5.97% ± 1.34% for those with very good dietary practice to 10.95% ± 1.6% for those with very poor dietary practice (*p* value: 0.006) [25]. Al-Lawati et al. reported that compared to estimated glomerular filtration rate (eGFR) below 60 ml/min/1.73 m^2^, the OR of good glycaemic control was 1.9 (*p* value 0.013) for those who have eGFR ≥ 60 ml/min/1.73 m^2^ [22]. Al Balushi et al. showed that compared to participants with HbA1c ≤ 7%, those with HbA1c > 7% had higher mean diastolic blood pressure (84 ± 9 mmHg compared to 80 ± 8 mmHg, *p* value: 0.006), higher total cholesterol (5.2 ± 1.3 mmol/l compared to 4.7 ± 0.8 mmol/l, *p* value: 0.002), and higher low-density lipoprotein (LDL) (3.8 ± 1.0 mmol/l compared to 3.0 ± 1.2 mmol/l, *p* value: 0.38) [14]. D'Souza et al. reported that a large proportion of those who completed diploma/technical degree had poor glycaemic control (67.4%) compared to those who completed high school (45.7%) and lower than high school (52.1%) (*p* value < 0.001) [15]. Two other studies did not find an association between education level and glycaemic control [10, 11]. The study by D'Souza et al. has also reported that patient perception of empowerment for self-management increased the likelihood of good glycaemic control (beta coefficient: 0.66, *p* value 0.001) [15].

In regard to the effect of modality of treatment on glycaemic control, Al-Nuaim et al. found that compared to participants on diet regimen, only the adjusted OR of poor control was 1.65 and 2.64, respectively, for those on oral agent and insulin [20]. Similarly, Al-Lawati et al. reported that the likelihood of good control was lower for those on oral agent (adjusted OR: 0.2, *p* value: 0.001) and for those on insulin (adjusted OR: 0.1, *p* value: 0.001) compared to diet regimen only [22]. Ajabnoor et al. reported, however, that participants on insulin had lower mean HbA1c (14.3 ± 1.0) compared to diet only (16.3 ± 1.8) and oral agent (17.0 ± 0.0) (*p* value < 0.001) [18].

## 4. Discussion

This systematic review summarises patient-related factors affecting glycaemic control among adults with T2DM in the GCC countries. Using a reproducible search strategy and prespecified inclusion/exclusion criteria, we identified 13 articles for inclusion in this review. The quality of the included studies is low to moderate in general, and the effect of some potentially important risk factors (including diet, physical activity, family support, and cognitive impairment) was not adequately investigated.

One cross-sectional study reported that age was associated with poor glycaemic control [15]. With advancing age, there might be a waning of the function of the *β*-cells of the pancreas, and some people develop other comorbidities that may affect glycaemic control. Similar to the findings of a previous systematic review [26], however, two of the included studies have found that elderly people with diabetes had better glycaemic control than young people [10, 22]. Elderly people are more likely to adhere to the management plan compared to young people who are more likely to be affected by the change in lifestyle and urbanisation [22, 27].

The duration of the disease is another important nonmodifiable risk factor of poor glycaemic control that was identified in three cross-sectional studies [15, 19, 22]. With longer duration of diabetes, the function of the pancreas further deteriorates because of the failure of the *β*-cells [28]. In addition, individuals with longer duration of diabetes are at a higher risk of developing diabetes-related complications, which can have a substantial effect on glycaemic control. With regard to the effect of gender on glycaemic control, two cross-sectional studies found an association but with contradicting results. One study reported that female gender was associated with poor glycaemic control [21], while another reported that females had better glycaemic control compared to males [22]. The inconsistency in the results of these studies may be explained in part by a variation in the methodology and heterogeneity between patients across the study population. In the systematic review undertaken by Sanal et al., however, it was found that the female gender was a risk of poor glycaemic control [26].

Compliance to diabetes management including adherence to diet, physical activity, medications, and self-monitoring of blood sugar is crucial in the management of diabetes. This current review shows that compliance to management and adherence to medications were significantly associated with good glycaemic control [19]. There is a gap in the knowledge, however, regarding barriers to compliance and adherence to management for people with diabetes in the GCC countries.

Two recent studies included in this review have found that compared to people with diet regimen only, those who were on oral agent and insulin had higher risk of poor control after adjustment for other risk factors [20, 22]. These findings may emphasize the potentially important role of lifestyle modification on the glycaemic control. In this review, however, we have found that the effect of lifestyle factors, including dietary habit and physical activity, on glycaemic control has not been studied adequately in the context of the GCC countries. Well-planned healthy eating habits with the supervision of a dietician can reduce HbA1c by 0.5 to 2.0% for people with T2DM [29–32]. There is also strong evidence of the effect of regular exercise on lowering HbA1c for people with T2DM [33–35]. Of the 13 studies included in this review, only two cross-sectional studies explored the association between dietary habits and glycaemic control [10, 25]. One study that assessed the association between various dietary practices and glycaemic control reported that consumption of carbonated (soda) drinks was the only associated factor [10]. The other study showed that participants who followed healthier dietary practices had a lower mean HbA1c [25]. Similarly, only two studies evaluated the association between physical activity and glycaemic control and reported no association [11]. In the latter four studies [10, 11, 25], however, the recruited sample was relatively small, no adjustment for confounders was done in most of them [11, 25], and the majority were of low to moderate [10, 25] quality.

Other modifiable risk factors that were found to be associated with glycaemic control in this review include patients' attitude to diabetes, the location of scheduled follow-up visits (hospital or primary healthcare centres), eGFR, anxiety, depression, diastolic blood pressure level, cholesterol level, LDL level, and patient empowerment for self-management [10, 14, 15, 19, 22, 24]. Studies included in this review, however, have assessed different sets of these risk factors; hence, a comparison of their findings was not possible.

In the GCC countries, wives are usually responsible for preparing meals for the whole family, including members with diabetes. For cultural reasons, on the other hand, some women prefer to be accompanied by a relative when they go out for exercise or when they visit healthcare centres. Therefore, it is likely that family support has an important role in the management of diabetes, which should be taken into account when evaluating glycaemic control. This is supported by the results of a systematic review showing a potential importance role of family support in the management of T2DM [36]. In addition, poorly controlled diabetes has been associated with a decline in cognitive function [37, 38], and impaired cognitve function is likely to have a negative effect on glycaemic control. That is because people with impaired cognition is less likely to be compliant to diabetes treatment plan.

Family support and cognitive function are potentially important risk factors for glycaemic control, which have not been explored in any of the included studies in this systematic review. Thus, the relationship of these factors with control needs a comprehensive investigation in future studies. Further, some of the modifiable risk factors such as the attitude towards the disease, barriers to compliance, anxiety, depression, and patient empowerment were not addressed adequately; hence, they need re-evaluation to build a stronger evidence. Future researchers should also reassess the association between glycaemic control and lifestyle factors using methodologically sound study design.

The strength of this review lies in the systematic, comprehensive, and unbiased approach taken during the literature search, data extraction, and assessment of the risk of bias. As the identified studies were either cross-sectional or case-control studies, a causal relationship between the risk factors and glycaemic control cannot be established. Moreover, because the studies in this review used different statistical methods, it was neither possible to generalise the magnitude of the effect of risk factors on glycaemic control nor possible to conduct a meta-analysis.

No studies assessing the factors affecting glycaemic control in Qatar or Kuwait were identified. Nevertheless, the results of this systematic review can be useful to all of the GCC countries. The populations of the GCC countries share similar cultures and lifestyles, and the healthcare services and medications are free of charge to all citizens.

## 5. Conclusion

This systematic review identified the following variables as the risk factors for poor glycaemic control in the GCC countries: low level of education, longer duration of diabetes, poor compliance to diet and medication, poor attitude towards the disease, poor self-management behaviour, anxiety, depression, renal impairment, hypertension, and dyslipidaemia. The policymakers should introduce large-scale awareness program and educational models to improve patient compliance and practices and to support patient empowerment for self-management. Healthcare providers should be aware of these risk factors and provide optimal care and guidelines for enriching self-management of the disease. The existing studies from the GCC have heterogeneity in their methodology, which may be related to the variation in their findings. In addition, some risk factors that may affect glycaemic control such as lifestyle, social support, and cognitive function have not been investigated adequately. Future research should address these issues.

## Figures and Tables

**Table 1:** Quality assessment tool for observational cohort and cross-sectional studies.

Criteria	Yes	No	Others (CD, NR, and NA)^∗^
(1) Was the research question or objective in this paper clearly stated?			
(2) Was the study population clearly specified and defined?			
(3) Was the participation rate of eligible persons at least 50%?			
(4) Were all the subjects selected or recruited from the same or similar populations (including the same time period)? Were inclusion and exclusion criteria for being in the study prespecified and applied uniformly to all participants?			
(5) Was a sample size justification, power description, or variance and effect estimates provided?			
(6) For the analyses in this paper, were the exposure(s) of interest measured prior to the outcome(s) being measured?			
(7) Was the time frame sufficient so that one could reasonably expect to see an association between exposure and outcome if it existed?			
(8) For exposures that can vary in amount or level, did the study examine different levels of the exposure as related to the outcome (e.g., categories of exposure or exposure measured as continuous variable)?			
(9) Were the exposure measures (independent variables) clearly defined, valid, reliable, and implemented consistently across all study participants?			
(10) Was the exposure(s) assessed more than once over time?			
(11) Were the outcome measures (dependent variables) clearly defined, valid, reliable, and implemented consistently across all study participants?			
(12) Were the outcome assessors blinded to the exposure status of participants?			
(13) Was loss to follow-up after baseline 20% or less?			
(14) Were key potential confounding variables measured and adjusted statistically for their impact on the relationship between exposure(s) and outcome(s)?			

Quality rating (good, fair, or poor) (see guidance)			
Rater no. 1 initials:			
Rater no. 2 initials:			
Additional comments (if poor, please state why):			

^∗^CD: cannot determine; NA: not applicable; NR: not reported.

**Table 2:** Quality assessment of case-control studies.

Criteria	Yes	No	Others (CD, NR, and NA)^∗^
(1) Was the research question or objective in this paper clearly stated and appropriate?			
(2) Was the study population clearly specified and defined?			
(3) Did the authors include a sample size justification?			
(4) Were controls selected or recruited from the same or similar population that gave rise to the cases (including the same time frame)?			
(5) Were the definitions, inclusion and exclusion criteria, algorithms, or processes used to identify or select cases and controls valid, reliable, and implemented consistently across all study participants?			
(6) Were the cases clearly defined and differentiated from controls?			
(7) If less than 100 percent of eligible cases and/or controls were selected for the study, were the cases and/or controls randomly selected from those eligible?			
(8) Was there use of concurrent controls?			
(9) Were the investigators able to confirm that the exposure/risk occurred prior to the development of the condition or event that defined a participant as a case?			
(10) Were the measures of exposure/risk clearly defined, valid, reliable, and implemented consistently (including the same time period) across all study participants?			
(11) Were the assessors of exposure/risk blinded to the case or control status of participants?			
(12) Were key potential confounding variables measured and adjusted statistically in the analyses? If matching was used, did the investigators account for matching during study analysis?			

Quality rating (good, fair, or poor) (see guidance)			
Rater no. 1 initials:			
Rater no. 2 initials:			
Additional comments (if poor, please state why):			

^∗^CD: cannot determine; NA: not applicable; NR: not reported.

**Table 3:** Included studies and their general features.

Author Publication year Country	Study design population	Population	Number of participants (male and female)	Age (mean age ± SD)	Glycaemic control measurement method(s)	Risk factors examined	Instrument used to measure risk factors
Ajabnoor 1987 [18] Saudi Arabia	Case-control	Diabetics attending King Abdulaziz University Hospital diabetic clinic	Cases 73 (23, 50) Controls 30 (19, 11)	—	HbA1c	Age, gender, FPG, and treatment modality	Questionnaire and lab test
Binhemd 1992 [19] Saudi Arabia	Cross-sectional	Females attending Diabetes and Endocrine Centre in Dammam	300 (0, 300)	42.6 ± 9.1	HbA1c	KAP (knowledge, attitude and practice), diabetes type, and diabetes duration	Interview using a questionnaire, anthropometric measures, and lab test
Al-Nuaim 1998 [20] Saudi Arabia	Cross-sectional	National (different regions of SA)	613 (320, 293)	Good control 47.0 ± 14.8 Poor control 51.5 ± 13.8	RBG	Age, BMI, region, residency, gender, and treatment modalities	Interview using a questionnaire, anthropometric measures, and lab test
Malik 1999 [24] United Arab Emirates	Cross-sectional	Patients attending Mafraq Hospital in Abu Dhabi and its surrounding health clinics	696	—	FPG	Age, gender, ethnicity, diabetes duration, family history, treatment modalities, and follow-up location	Interview using a questionnaire and lab test
Abdelmoneim 2002 [21] Saudi Arabia	Cross-sectional	Patients attending diabetic clinic in a primary healthcare centre	198 (90, 108)	Males 59.5 ± 8.9 Females 53.8 ± 8.1	FPG	Age, gender, family history, diabetes duration, BMI, cholesterol level, complications, number of health education session, and crowding index	Review of medical records
Al-Kaabi 2008 [10] United Arab Emirates	Cross-sectional	Diabetic patients attending outpatient clinic at Tawam Hospital and primary healthcare centres in Al Ain district	409 (158, 251)	51.4 ± 11.2	HbA1c	Age, gender, marital status, level of education, occupation, smoking, eating practice, BMI, abdominal circumference, blood pressure, and lipid profile	Interviewer-administered questionnaire and anthropometric measures. Blood tests from medical records
Al-Kaabi 2009 [11] United Arab Emirates	Cross-sectional	Diabetic patients attending outpatient clinic at Tawam Hospital and primary healthcare centres in Al Ain district	309 (68, 241)	52 ± 9.9	HbA1c	Age, gender, nationality, marital status, level of education, employment, income, diabetes duration, smoking, diabetes complications, physical activity, BMI, abdominal circumference, and blood pressure	Interviewer-administered questionnaire and anthropometric measures. Blood tests from medical records
Al-Lawati 2012 [22] Oman	Cross-sectional	Multicentred (different regions of Oman)	1266 (570, 696)	53.3 ± 11.5	HbA1c	Age, gender, duration of diabetes, treatment modalities, BMI, eGFR, smoking, and healthcare index	Data collected from patients' medical records
Al-Hayek 2012 [8] Saudi Arabia	Cross-sectional	Patients attending Sultan Bin Abdulaziz Humanitarian City, Riyadh	147 (99, 48)	57.3 ± 14.4	HbA1c	Age, gender, marital status, employment, BMI, self-care management behaviour, self-monitoring of blood glucose, medication adherence, anxiety, and depression	Interviewer-administered questionnaire and anthropometric measures. Blood tests from medical records
Shamsi 2013 [25] Bahrain	Cross-sectional	Patients attending 5 healthcare centres in Bahrain	400 (192, 208)	54.7 ± 9.95	HbA1c	Dietary practice	Interviewer-administered uestionnaire. Anthropometric measures and blood tests results were collected from medical records
Al Balushi 2014 [14] Oman	Cross-sectional	Type 2 patients attending Al Dakhliya region primary healthcare centre	177 (71, 106)	53 ± 12	FBG HbA1c	Age, gender, diabetes duration, BMI, blood pressure, creatinine, and lipid profile	Data collected from patients' medical records
Alrahbi 2014 [23] Oman	Cross-sectional	Patients attending 35 healthcare centres in Muscat and Al Dakhliya	266 (121, 145)	—	HbA1c	Diabetes self-management	Self-administered questionnaire and blood test for HbA1c
D'Souza 2015 [15] Oman	Cross-sectional	Patients attending diabetic clinic at a public hospital in Oman	300 (143, 157)	—	HbA1c	Age, gender, education, diabetes duration, diabetes education, medication, and BMI	Interviewer-administered questionnaire and patient's medical records for HbA1c

HbA1c: haemoglobin A1c; FPS: fasting plasma sugar; eGFR: estimated glomerular filtration rate; TTT: treatment.

**Table 4:** Risk of bias for the included studies.

Study	Criteria 1	Criteria 2	Criteria 3	Criteria 4	Criteria 5	Criteria 6	Criteria 7	Criteria 8	Criteria 9	Criteria 10	Criteria 11	Criteria 12	Criteria 13	Criteria 14	Overall quality
Case-control															
Ajabnoor 1987 [18]	Yes	Yes	No	No	NR	Yes	No	NR	Yes	Yes	No	No	—	—	Low
Cross-sectional															
Binhemd 1992 [19]	Yes	Yes	NR	Yes	No	NA	NA	No	Yes	NA	Yes	NA	NA	No	Low
Al-Nuaim 1998 [20]	Yes	Yes	NR	Yes	No	NA	NA	Yes	Yes	NA	Yes	NA	NA	Yes	High
Malik 1999 [24]	Yes	No	NR	Yes	No	NA	NA	Yes	Yes	NA	Yes	NA	NA	No	Low
Abdelmoneim 2002 [21]	Yes	Yes	NR	Yes	No	NA	NA	No	Yes	NA	Yes	NA	NA	Yes	Moderate
Al-Kaabi 2008 [10]	Yes	Yes	NR	Yes	No	NA	NA	No	No	NA	Yes	NA	NA	Yes	Moderate
Al-Kaabi 2009 [11]	Yes	Yes	NR	Yes	No	NA	NA	Yes	Yes	NA	Yes	NA	NA	No	High
Al-Lawati 2012 [22]	Yes	Yes	NR	Yes	No	NA	NA	Yes	Yes	NA	Yes	NA	NA	Yes	Moderate
Al-Hayek 2012 [8]	Yes	Yes	NR	Yes	No	NA	NA	Yes	Yes	NA	Yes	NA	NA	No	Low
Shamsi 2013 [25]	Yes	Yes	NR	Yes	No	NA	NA	Yes	Yes	NA	Yes	NA	NA	No	Moderate
Al Balushi 2014 [14]	Yes	Yes	NR	Yes	No	NA	NA	Yes	Yes	NA	Yes	NA	NA	No	Low
Alrahbi 2014 [23]	Yes	Yes	NR	Yes	No	NA	NA	Yes	Yes	NA	Yes	NA	NA	No	Low
D'Souza 2015 [15]	Yes	Yes	NR	Yes	Yes	NA	NA	Yes	Yes	NA	Yes	NA	NA	No	Low

NR: not reported; NA: not applicable.

**Table 5:** Results of the included studies.

Author Year	Risk factors examined	Risk factors associated with glycaemic control	Statistical analysis of positive results					Main conclusion
Ajabnoor 1987 [18]	Age, gender, duration of diabetes, FPG, and treatment modality	FPG and treatment modalities	FPG/HbA1c correlation *r* = 0.19, *p* < 0.005					HbA1c is associated with FBG and treatment modality but not with age, gender, and duration of diabetes
			Treatment modality	Mean HbA1c		*p* value		
			Insulin	14.3 ± 1.3				
			Oral	17.0 ± 0.0		<0.001		
			Diet	16.3 ± 1.8		0.02		

Binhemd 1992 [19]	Knowledge, attitude and practice (KAP), diabetes type, and diabetes duration	KAP and diabetes duration	Positive correlation between HbA1c and diabetes duration (*p* < 0.001). Negative correlation between HbA1c and the practice (*p* : 0.03) and the attitude (*p* : 0.01)					The positive correlation between HbA1c and the patients' practice and attitude reflects the need for continuous patient education, follow-up, and support

Al-Nuaim 1998 [20]	Age, BMI, region, residency, gender, and treatment modalities	TTT modalities	Adjusted odds ratio and 95% confidence interval (95% CI) of poor glycaemic control					There is a significant relation between glycaemic control and treatment modalities
			TTT modality (ref: diet regimen)					
				Odds ratio		95% CI	*p* value	
			Oral agent	1.7		1.1–2.6	0.005	
			Insulin	2.6		1.4–5.0		

Malik 1999 [24]	Age, gender, ethnicity, diabetes duration, family history, treatment modalities, and follow-up location	Duration of diabetes, follow-up location, and family history	Control			Odds ratio (*p* value)		Improvements are needed in primary care and in the community-based approach to diabetes control
						Fair	Poor	
			Diabetes duration (years)			1.03 (0.329)	1.08 (0.007)	
			Follow-up at health centres (ref: hospital)			1.86 (0.036)	2.47 (0.001)	
			Family history (ref: none)			0.62 (0.095)	0.39 (0.001)	

Abdelmoneim 2002 [21]	Age, gender, family history, diabetes duration, BMI, cholesterol level, complications, number of health education session, and crowding index	Gender and health education	Odds ratio (*p* value) (ref: good control)					Female sex is a significant predictor of poor glycaemic control, and among females, the lower the number of education sessions, the poorer the diabetes control
			Gender (ref: male)	2.84 (<0.05)				
			Health education among females	0.28 (<0.05)				
			Health education among males	1.39 (<0.05)				
Al-Kaabi 2008 [10]	Age, gender, marital status, level of education, occupation, smoking, eating practice, BMI, abdominal circumference, blood pressure, and lipid profile	Carbonated drinks age	Regression analyses of HbA1c level (adjusted beta coef. (*p* value))					The dietary practice of people with diabetes in the UAE is inadequate and needs improvement
			Number of carbonated drinks	0.201 (0.029)				
			Age	0.023 (0.047)				

Al-Kaabi 2009 [11]	Age, gender, nationality, marital status, level of education, employment, income, diabetes duration, smoking, diabetes complications, physical activity, BMI, abdominal circumference, and blood pressure	No significant association	Multiple regression analysis of HbA1c in relation to age, gender, education, duration of diabetes, and physical activity did not reveal any significant association					The physical activity practice of people with diabetes in the UAE is largely inadequate to meet the recommendations

Al-Lawati 2012 [22]	Age, gender, duration of diabetes, treatment modalities, BMI, eGFR, smoking, and healthcare index	Age, gender, eGFR, diabetes duration, and TTT modalities	Adjusted odds ratio and *p* value of good glycaemic control					Younger Omani adults exhibit worse glycaemic levels compared to older adults
			Age (ref: 20–39 yrs)		40–59 yrs	1.7	0.01	
					60+ yrs	2.5	0.0001	
			Sex (ref: men)		Women	1.5	0.001	
			Diabetes duration (ref: <5 yrs)		≥5 yrs	0.8	0.041	
			Treatment type (ref: diet)		Oral	0.2	0.001	
					Insulin ± oral	0.1	0.001	
			eGFR (ref: <60 ml/min/1.73 m^2^		≥60 ml/min	1.9	0.001	

Al-Hayek 2012 [8]	Age, gender, marital status, employment, BMI, self-care management behaviour, self-monitoring of blood glucose, medication adherence, anxiety, and depression	Medication adherence, anxiety, and depression			HbA1c < 7%	HbA1c ≥ 7%	*p* value	Poor diabetes self-care management behaviour, low adherence to medicine, and higher level of anxiety and depression are associated with poor glycaemic control
			Medication adherence		7.4 ± 1.4	5.4 ± 1.2	0.0007	
			Anxiety		7.9 ± 1.3	10.3 ± 1.7	0.0005	
			Depression		6.9 ± 0.9	9.8 ± 1.3	0.0002	
			Total hospital anxiety and depression scale (HADS)		14.8 ± 1.8	20.1 ± 2.1	0.0001	

Shamsi 2013 [25]	Dietary practice	Dietary practice	Dietary practice		HbA1c (mean ± SD)		*p* value	There is a significant relation between the dietary practice and the HbA1c level
			Very poor		10.95 ± 1.56		0.006	
			Poor		7.46 ± 1.74			
			Average		7.46 ± 1.97			
			Good		7.31 ± 2.07			
			Very good		5.97 ± 1.36			
Al Balushi 2014 [14]	Age, gender, diabetes duration, BMI, blood pressure, creatinine, and lipid profile	Total cholesterol, diastolic blood pressure, and LDL			HbA1c < 7%	HbA1c ≥ 7%	*p* value	There is a significant association between HbA1c and diastolic blood pressure, total cholesterol, and LDL
			Diastolic blood pressure, mmHg (mean ± SD)		80 ± 8	84 ± 9	0.006	
			Total cholesterol, mmol/l (mean ± SD)		4.7 ± 0.8	5.2 ± 1.3	0.002	
			LDL, mmol/l (mean ± SD)		3.0 ± 1.2	3.8 ± 1.0	0.034	

Alrahbi 2014 [23]	Diabetes self-management	No association between diabetes self-management and glycaemic control was found	No association was found					There is no association between diabetes self-management and glycaemic control

D'Souza 2015 [15]	Age, gender, education, diabetes duration, diabetes education, medication, BMI, and patient empowerment	Age, education, diabetes duration prior to diabetes education, TTT modalities empowerment, effect of diabetes on activities of daily living			HbA1c < 7%	HbA1c ≥ 7%	*p* value	Interventions to increase the empowerment of people with T2DM should be made for better glycaemic control
			Age (no. (%))	30–39 yrs	24 (51.1)	23 (48.9)	0.000	
				40–49 yrs	52 (50.5)	51 (49.5)		
				50–59 yrs	36 (39.1)	56 (60.9)		
				≥60 yrs	26 (44.8)	32 (55.2)		
			Education (no. (%))	Until 8th grade	56 (47.9)	61 (52.1)	0.000	
				High school	51 (54.3)	43 (45.7)		
				Diploma/tech	31 (10.3)	58 (67.4)		
			Duration of diabetes (no. (%))	0–9 yrs	57 (50.9)	55 (49.1)	0.000	
				10–19 yrs	68 (47.2)	76 (52.8)		
				≥20 yrs	13 (29.5)	31 (70.5)		
			Diabetes education program (no. (%))	No	54 (47.0)	61 (53.0)	0.000	
				Yes	84 (45.4)	101 (54.6)		
			Medications (no. (%))	Oral	109 (50.5)	107 (49.5)	0.000	
				Insulin and oral	29 (34.5)	55 (65.5)		
			Regression analysis					
			Empowerment and glycaemic control		Beta coef.	*p* value		
					0.657	0.001		

HbA1c: haemoglobin A1c; FPS: fasting plasma sugar; eGFR: estimated glomerular filtration rate; TTT: treatment; ref: reference.
